# Interaction of Recombinant *Gallus gallus* SEPT5 and Brain Proteins of H5N1-Avian Influenza Virus-Infected Chickens

**DOI:** 10.3390/proteomes5030023

**Published:** 2017-09-12

**Authors:** Jasmine Elanie Khairat, Vinod Balasubramaniam, Iekhsan Othman, Abdul Rahman Omar, Sharifah Syed Hassan

**Affiliations:** 1 Jeffrey Cheah School of Medicine and Health Sciences, Monash University Malaysia, Jalan Lagoon Selatan, Bandar Sunway, Subang Jaya 47500, Selangor, Malaysia; jasmine.e.khairat@gmail.com (J.E.K.); vinod.balasubramaniam@monash.edu (V.B.); iekhsan.othman@monash.edu (I.O.); 2 Infectious Diseases and Health, Tropical Medicine and Biology Platform, Jeffrey Cheah School of Medicine and Health Sciences, Monash University Malaysia, Bandar Sunway, Subang Jaya 47500, Selangor, Malaysia; 3 Institute of Bioscience, Faculty of Veterinary Medicine, University Putra Malaysia, Serdang 43400, Selangor, Malaysia; aro@upm.edu.my

**Keywords:** septins, septin5, H5N1 avian influenza virus, co-immunoprecipitation, chicken brain

## Abstract

Septin forms a conserved family of cytoskeletal guanosine triphosphate (GTP) binding proteins that have diverse roles in protein scaffolding, vesicle trafficking, and cytokinesis. The involvement of septins in infectious viral disease pathogenesis has been demonstrated by the upregulation of SEPT5 protein and its mRNA in brain tissues of H5N1-infected chickens, thus, providing evidence for the potential importance of this protein in the pathogenesis of neurovirulence caused by the avian influenza virus. In this study, cloning, expression, and purification of *Gallus gallus* SEPT5 protein was performed in *Escherichia coli.* The *SEPT5* gene was inserted into the pRSETB expression vector, transformed in the *E. coli* BL21 (DE3) strain and the expression of SEPT5 protein was induced by IPTG. The SEPT5 protein was shown to be authentic as it was able to be pulled down by a commercial anti-SEPT5 antibody in a co-immunoprecipitation assay. In vivo aggregation of the recombinant protein was limited by cultivation at a reduced temperature of 16 °C. Using co-immunoprecipitation techniques, the purified recombinant SEPT5 protein was used to pull down host’s interacting or binding proteins, i.e., proteins of brains of chickens infected with the H5N1 influenza virus. Interacting proteins, such as CRMP2, tubulin proteins, heat-shock proteins and other classes of septins were identified using LCMS/MS. Results from this study suggest that the codon-optimized SEPT5 gene can be efficiently expressed in the *E. coli* bacterial system producing authentic SEPT5 protein, thus, enabling multiple host’s proteins to interact with the SEPT5 protein.

## 1. Introduction

Septins are a family of GTPases which are found in the cytosol. Septins are broadly expressed throughout the animal kingdom; however, these 40–50 kDa proteins are well conserved between different species. The human septin family is composed of 14 loci, SEPT1–SEPT14 which encodes the different septin proteins [1]. The primary structure of septins is characterized by variable N- and C-termini and a conserved central domain. In the central domain, a P-loop signature allocates septins in the P-loop GTPase family. Directly adjacent to the variable N-terminal tail is a short polybasic sequence that has been found to be responsible for binding of septins to hosts’ membrane phospholipids [2]. This is followed by the GTP-binding domain that spans most of the septin sequence. All septins, with the exception of the SEPT3 group, also contain a coiled-coil region right before the variable C-terminal tail [3]. The N- and C-termini surrounding the core domain of the septins are divergent and some of the mammalian septins have a predicted C-terminal coiled-coil domain which could be involved in protein-protein interactions [4,5,6]. There also exist two conserved regions in septins that are involved in the formation of septin–septin interactions and also complex assembly; the guanine nucleotide-binding domain (G interface), and the N- and C-terminal extensions (NC interface) [7]. Based on sequence similarities, septins can be grouped into four subgroups. SEPT5, together with SEPT1, SEPT2, and SEPT4, belongs to the SEPT2 group or group III. Septins have been associated in multiple cellular functions, including membrane transport, apoptosis, cell polarity, cell cycle regulation, cytokinesis, and oncogenesis [8,9,10].

Apart from formation of hetero-oligomeric complexes, septins from different groups also interact and associate with cellular membranes, actin filaments, and microtubules [11]. They are also dispersed throughout the cytoplasm without forming a continuous polymer network and served as basins or links that sequester key signalling molecules with remote functions [12]. One example is the SEPT2/6/7 complex, which acts as scaffold for microtubules-associated-protein 4 (MAP4). MAP4 is one of the ubiquitous MAPs that stabilize microtubules by bundling. By binding to the septin complex, MAP4 cannot interact with microtubules, whereby the septin complex sequesters the MAP4 activity away from microtubules, essentially acting as a basin. By depleting one of the subunits, it resulted in hyper-stabilization of the microtubules by releasing MAP4 from the septin depository. Thus, the septin scaffolds dispersed in the cytoplasm could indirectly control the stability and dynamicity of the microtubules.

Expression and localization of different septins in the human brain have been studied by Kim et al. in 2004, which showed variable levels and expression patterns of the different septins in brain tissue, brain tumour specimens, and human brain tumour cell lines [13]. In human brains, the possible roles for septins in neurologic disorders have emerged based on the discovery of the brain-specific expression of some septins in different parts of the brains and its interaction with key components that are related to the disease. In a study of yeast two hybrids, it was demonstrated that SEPT6 interacts with transmembrane myelin protein, other than being associated with multiple septin complexes, SEPT2 and SEPT7, to form the cytoskeleton in myelinating glia [14]. This suggests the critical dependence of septins’ arrangements into complexes for proper structure formation and functions. SEPT2 (NEDD5), SEPT1 (DIFF6), and SEPT4 (H5) have been found to be associated with Alzheimer-specific neuro fibrillar tangles by use of immunocytochemistry and immunoelectron microscopy [15], while co-immunoprecipitation assay revealed SEPT5 (CDCRel-1) interact with Parkin, a pathogenic protein in Parkinson’s Disease [16]. SEPT11 plays a functional role in the cytoarchitecture of neurons and GABAergic synaptic connectivity and is present in GABAergic synapses. The regulation of cytoarchitecture of neurons also involved another family of septin which is SEPT6 [17]. SEPT3, on the other hand, is a developmentally-regulated phosphoprotein enriched in presynaptic nerve terminals [18]. Mammalian SEPT5 was discovered to be localized to plasma membranes and highly concentrated at the tips of the neurites where it associates with SNARE complex, which regulates vesicle targeting and fusion, thus inhibiting exocytosis [19]. It also co-purified with other proteins related with exocytosis, such as syntaxins, synaptosomal-associated protein 25 (SNAP-25), and synaptophysin [20]. In addition, rat brain septin CDC10 also co-immunoprecipitated with sec6/8 complexes which are involved in vesicle delivery [21]. All septins also interacted with other non-septin proteins.

Recently, it was shown that some pathogens use septins linked to several viral-encoded proteins to their advantage. The replication of hepatitis C virus (HCV) which is a positive-strand RNA virus that causes chronic hepatitis [22], for example, requires the host-encoded hnRNPA1, an RNA-binding protein, that, together forms a quaternary complex with viral RNA, NS5b, and host-encoded SEPT6. The loss of any of these host components resulted in a reduction in viral reproduction. Another virus, KSHV, or Kaposi’s sarcoma-associated herpesvirus, was reported to bind to SEPT4 proteins [23]. SEPT4 play a role in promoting apoptosis of the infected cells and the Kaposin A-SEPT4 interaction apparently blocks this pro-apoptotic effect, enhancing tumourigenesis of KSHV in KSHV-infected cells.

In the present study, we expressed the purified and co-immunoprecipitated authentic recombinant SEPT5 protein of chicken for interaction studies with brain lysates from chickens infected with H5N1 avian influenza virus and non-infected chickens. In the infected brains, proteins, such as dihydropyrimidinase-related protein 2, heat shock proteins and other septins were identified as interacting partners of the SEPT5 recombinant protein which might affect virus pathogenesis. These data will be indispensable for future studies to understand the role of SEPT5 in H5N1 virus infection.

## 2. Materials and Methods

### 2.1. H5N1-Infected and Non-Infected Brain Tissues and Paraffin Blocks

Highly-pathogenic avian influenza (HPAI) H5N1-infected and non-infected formalin fixed paraffin embedded brain tissues and wet brain tissues (stored at −80 °C in a BSL-3 facility of the Institute of Bioscience) were archived samples and provided by Prof Abdul Rahman Omar, UPM. Briefly, four-week old SPF chickens were infected with HPAI H5N1 virus (A/Chicken/Malaysia/5858/04: NCBI Taxon ID: 365120) as a virus control group in a vaccine trial and the brains of the virus-infected and control birds were harvested. The A/Chicken/Malaysia/5858/04 virus was previously been tested to be highly pathogenic, as over 75% mortality was observed in four- to eight-week-old chickens following an intravenous pathogenicity test. The brains were also tested to be free of Newcastle Disease virus. All work involving the use of wet brain tissues infected with this live H5N1 virus was conducted in the BSL-3 laboratory facility of the Institute of Bioscience, UPM.

### 2.2. Immunohistochemistry Staining for the Detection of H5N1 Virus and SEPT5 in Brain of Infected Chickens

The formalin-fixed paraffin embedded blocks of infected and non-infected chicken brains were sectioned for immunoperoxidase assay (IPA) and hematoxylin and eosin (H and E) staining with manual microtome (Histology Labs., Faculty of Veterinary Science, UPM). The paraffin blocks were processed for immunohistochemistry staining as previously described by Brown et al. [24] with modifications. For the IPA method, the tissue sections from H5N1-infected and control chickens were deparaffinized in xylene and hydrated with graded alcohol. Hydrated samples were then subjected to antigen retrieval with EDTA (in buffer: 1.0 M, 0.05% Tween-20) at 95 °C–100 °C in steaming water bath. The slides were then treated with 3% hydrogen peroxide in methanol at room temperature and blocked with normal horse serum at 37 °C in a humidified chamber. Slides were incubated with mouse polyclonal anti-SEPT5 (1:200) (Abcam, Cambridge, MA, USA) or rabbit monoclonal anti-NP H5N1 (Santa Cruz Biotechnology, Inc., Dallas, TX, USA), overnight in 4 °C. The slides were then incubated with biotin-conjugated UNIVERSAL anti-mouse/anti-rabbit IgG secondary antibody for 1 h at 37 °C. Avidin/biotin blocking was done using VECTASTAIN^®^ Universal Quick Kit, R.T.U (Vector Laboratories, Burlingame, CA, USA) as per the manufacturer’s protocol. Slides were flooded with the avidin-biotin complex solution for 30 min at room temperature and stained with DAB (3,3′-Diaminobenzidine) solution. The immunoperoxidase and H and E-stained slides were observed under compound light microscope for visualization of histopathological changes of the tissues.

### 2.3. Recombinant SEPT5 Protein Production in an E. coli Expression System

#### 2.3.1. Design of SEPT5 *Gallus gallus* Gene Primers and Restriction Enzyme Sites

Nucleotide and amino acid sequences of SEPT5 *Gallus gallus* were obtained from the NCBI database. The start and end of SEPT5 was chosen based on conserved regions mapped by Pfam database [25] and other alignments produced with the amino acid sequence of *Gallus gallus* SEPT5 (ncbi ID: 001025825.2). Oligonucleotides were synthesized for the amplification of SEPT5 based on the SEPT5 sequence ID. Using both the cDNA sequence, specific PCR primers were designed by Primer3Plus 2.0 software [26] to amplify the corresponding gene as shown in Table 1.

Restriction enzyme sites *Eco*RI and *Hind*III were added to the sense and antisense primers to facilitate subsequent cloning into pTZ57R/T (Thermo Scientific, Life Technology, Waltham, MA, USA) vector and pRSETB (Invitrogen, Carlsbad, CA, USA) expression vectors. Expression was induced by the promoter T7 RNA polymerase in BL21 (DE3) *E. coli* cells.

#### 2.3.2. RNA Extractions from Brain Tissues

RNA of H5N1-infected brain tissues (provided by Prof. Abdul Rahman Omar) was prepared using standard RNA extraction techniques. Total RNA was prepared from tissues using a combination of Qiagen RNeasy Mini Kit (Qiagen, Valencia, CA, USA) following the manufacturer’s instructions and TRIZOL extraction protocol. Purified RNA was eluted in RNAse-free water and stored in −80 °C until further use. Primary experimental procedure involved whole RNA extraction and amplification of the gene with the first strand cDNA as the template.

#### 2.3.3. First Strand cDNA Synthesis

Total RNA from the brain lysates were used as a template for Random Primers (Promega, San Luis Obispo, CA, USA) first-strand cDNA synthesis using reverse transcriptase (Promega). The reverse transcription reaction mixtures contained total RNA (5 μg), dNTP, primers, and RNase-free water. Reaction mixture was incubated at 70 °C for 10 min; the reverse transcription was carried out in the presence of AMV reverse transcriptase and RNase inhibitor at 42 °C for 50 min. The cDNAs were kept at 20 °C for further use. PCR was carried out to amplify SEPT5 with its specific sense and antisense primers along with annealing temperatures of 58 °C and an extension time at 35 cycles.

#### 2.3.4. Cloning of SEPT5 *Gallus gallus* into the pTZ57R/T Vector

PCR products were detected by ethidium bromide (EtBr) staining after TAE-1% agarose gel electrophoresis, purified with a Wizard SV Gel/PCR Clean-Up kit (Promega, San Luis Obispo, CA, USA), cloned into the pTZ57R/T (Thermo Scientific, Life Technology, Waltham, MA, USA) vector according to manufacturer’s instructions, and transformed into DH5α *E. coli* strains. Transformation was done with heat shock treatment of bacterial cells at 42 °C and cells were left to grow overnight at 37 °C. Plasmid DNA was extracted from bacterial culture and purified with Zyppy™ Plasmid Miniprep Kit (ZYMO Research Corp, Orange, CA, USA) as per the manufacturer’s protocol.

#### 2.3.5. SEPT5 Protein Expression in *E. coli*

To express the SEPT5 protein of *Gallus gallus*, both the purified DNA of SEPT5 from the cloning vector and expression vector, pRSETB were double digested with *Eco*RI *and Hind*III enzymes and ligated with T4 DNA ligase overnight at 4 °C. Recombinant pRSETB-SEPT5 plasmids were transformed into *E. coli* BL21 (DE3) cells with His-tag. The bacterial plasmid was induced with 0.2 mM IPTG for 18 h at 16 °C to express the SEPT5 recombinant proteins. Cells were collected and sonicated in cold lysis buffer (10 mM Tris–HCl, 0.3 M NaCl, 1 mM EDTA, 1 mM phenylmethylsulfonylfluoride (PMSF), pH 7.5). After centrifugation at 12,000× *g* for 20 min, the supernatant was recovered.

#### 2.3.6. SEPT5 Protein Purification with His-Tag Column

Crude septin proteins obtained from supernatant of lysed BL21 (DE3) cells were applied to a Ni^+^-NTA His-tag column (Novagen, Birmingham, UK) and the column was washed with wash buffer containing 50 mM phosphate, 0.3 M NaCl, and 20 mM imidazole (pH 8.0). Proteins were then eluted with elution buffer containing 50 mM phosphate, 0.5 M NaCl, and 0.5 M imidazole (pH 8.0). Protein fractions from the supernatants, flow-through, washes and, finally, purified SEPT5 were detected by 12% SDS–PAGE followed by Coomassie Blue staining. Western blotting of purified recombinant SEPT5 (rSEPT5) was detected using alkaline-phosphatase system (Promega, San Luis Obispo, CA, USA) with commercially-available anti-SEPT5 polyclonal antibodies (Abcam, Cambridge, MA, USA). Briefly, the SDS-PAGE gel was transferred to a PVDF membrane (Millipore, Billerica, MA, USA) by semi-dry electroblotting (Biorad, Hercules, CA, USA). The membrane was blocked with blocking buffer for 90 min at room temperature and washed with washing buffer for 10 min at room temperature. The membrane was probed with an anti-SEPT mouse polyclonal antibody (Abcam) overnight at 4 °C followed by incubation with goat anti-mouse antibody (Santa Cruz, CA, USA) at room temperature. Visualization of proteins was achieved using BCIP (5-bromo-4-chloro-3-indolyl-phosphate) in conjunction with NBT (nitro blue tetrazolium) for the colorimetric detection of alkaline phosphatase activity at 1–4 min exposure time.

### 2.4. Co-Immunoprecipitation Assay of rSEPT5 with Polyclonal Anti-SEPT5 Antibodies

An immunoprecipitation assay was conducted to determine that the eluted purified rSEPT5 protein is in its conformational state. In this assay, 25 μg of anti-SEPT5 polyclonal antibodies (Abcam, Cambridge, MA, USA) were added to 50 μL of Protein A/G agarose beads for 4 h at room temperature. The purified recombinant SEPT5 proteins in the elution buffer were added to the bound antibodies-bead complex followed by overnight incubation at 4 °C. The immunocomplexes were washed with wash buffer (150 mM NaCl, protease inhibitor cocktail) and the antigen antibody-complex was eluted from the beads by heating in SDS PAGE sample buffer. The eluted protein was analysed by SDS PAGE gel and MS/MS.

### 2.5. Pull-Down of Interacting Proteins of SEPT5 in Brain Lysates by Co-Immunoprecipitation

#### 2.5.1. Preparation of Chicken Brain Tissue Homogenates

Chicken brain homogenates were prepared for the purpose of interaction studies in a co-immunoprecipitation (co-IP) assay. Cytosolic soluble fractions and insoluble membrane fractions were extracted according to the procedure previously described with some modifications [27]. Upon thawing, the H5N1-infected and non-infected brains were weighed and recorded. The brains were then ground and homogenized with Dounce homogenizer at 4 °C in lysis/wash buffer (0.5 M Tris-HCl, 150 mM NaCl, 0.1 mM EDTA, 1% NP-40, 5% glycerol; pH 7.4) with a cocktail of PMSF protease inhibitors. These tissue homogenates were centrifuged at 12,000 rpm for 20 min at 4 °C. Cell lysates were aspirated and re-centrifuged for another 20 min. After a few rounds of centrifugation to obtain clear lysates, they are stored in −80 °C for long-term storage. Concentrations of lysates were determined by A280 UV absorbance value.

#### 2.5.2. Pull-Down of Interacting Proteins by co-IP

Co-IP study was performed as detailed by Corti et al. [28]. Briefly, antibodies immobilization complex was prepared by binding 25 μL protein A/G agarose resin (Pierce^®^) to anti-SEPT5 polyclonal antibody (Abcam, Cambridge, MA, USA) at room temperature. Prior to co-IP, ControlAmino Resin Link was added to the infected brain or non-infected brain protein lysates and purified rSEPT5 protein for 1 h, 4 °C to pre-clear non-specific binding proteins. Samples were centrifuged and supernatants were used for the study. To each pre-cleared uninfected and infected samples, immune complexes were added, followed by an overnight shaking incubation at 4 °C. The negative control consisted of quenching buffer with the agarose resin beads. Samples were with 1 M NaCl. After a final wash and centrifugation, eluted samples were re-suspended in protein loading buffer, heat denatured at 95 °C, centrifuged and resolved in 12% SDS-PAGE electrophoresis. Gel was then stained with silver stain according to the manufacturer’s protocol. Proteins were digested in solution with trypsin and sent for MS/MS analysis.

## 3. Results

### 3.1. Virus Pathogenicity of H5N1 Virus in Chicken Brains

**Figure 1:**
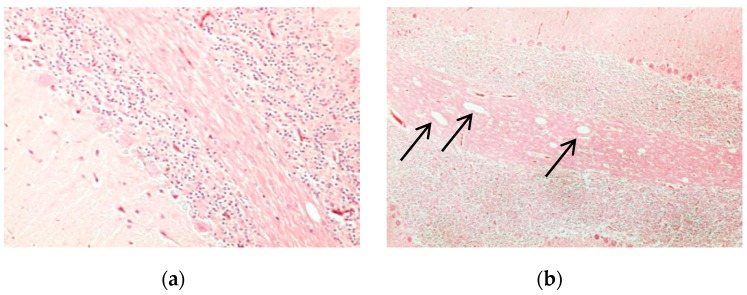
Histological analysis of cerebellum of chicken brain tissues as observed by H and E staining. Compared to non-infected animals (**a**), arrows indicate characteristics of coalescing areas of spongiosis in the white matter of the cerebellum that were observed in infected chickens (**b**). Image was viewed at 200× magnification.

**Figure 2:**
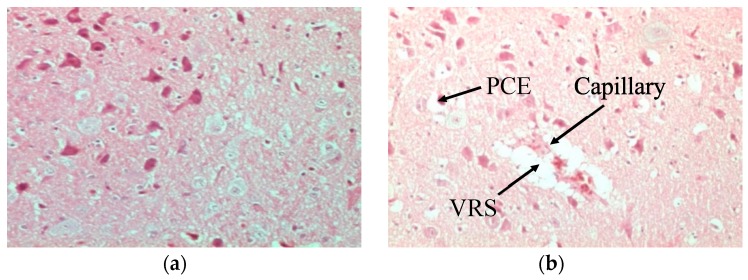
Images of cerebral tissues showed oedema in infected chickens as observed by H and E staining at 200× magnification. No widening of pericellular or perivascular space was seen in non-infected tissues (**a**). Infected tissues showed pronounced widening of the pericellular space around the cortical neurons indicating pericellular oedema. Additionally, a cortical capillary blood vessel with pronounced widening of the Virchow Robin space is seen (**b**).

**Figure 3:**
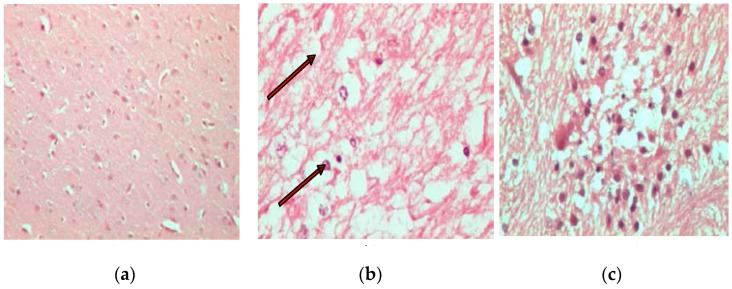
Histological images of chicken cerebral tissues showing obvious vacuolation as observed in H and E staining. Arrows indicated areas of intense vacuolation in infected tissue (**b**) at 400× magnification compared to non-infected tissues (**a**). A large amount of multifocal tissue necrosis in infected brain occurred along with high recruitment of inflammatory cells around necrotic tissues (**c**).

In comparison to normal non-infected brains of chickens stained with H and E (Figure 1a), the brains of H5N1-infected chickens presented marked histological changes, such as spongiosis in the white matter of the cerebellum (Figure 1b). Normal tissues did not show any generalized ooedema (Figure 2a), as observed in infected brain tissues (Figure 2b). Deterioration of brain tissues due to infection with HPAI H5N1 infection can be seen in Figure 3b,c when compared to healthy tissues (Figure 3a).

### 3.2. Localization of SEPT5 Proteins in Chicken Brains

**Figure 4:**
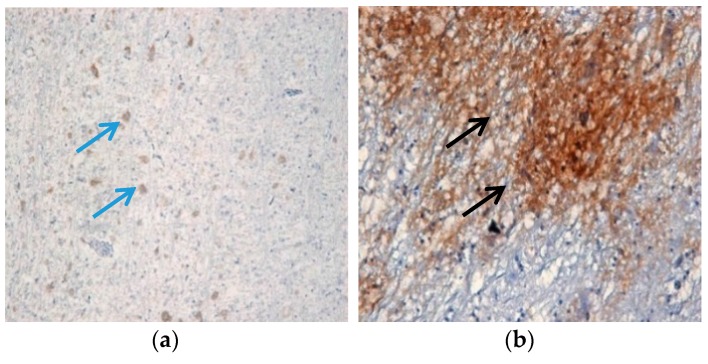
Septin proteins in brain tissues. Septin proteins were found in the brain tissues of uninfected and infected tissues. (**a**) Slight brown staining was seen in healthy chicken brain at 20× magnification in comparison to infected tissues with intense brown staining observed at 40× magnification (**b**).

To determine the presence and absence of SEPT5 proteins in chicken brain tissues, both infected and uninfected brain samples were tested for SEPT5 with anti-SEPT5 antibodies. In both tissues, septin was present in the substantia region of the brain. In infected tissues, the brown stain was visible surrounding the degenerated and necrotic tissues (Figure 4a) and septin was also present in the normal uninfected neuronal cells (Figure 4b). However an intense brown stain in infected tissues indicated an abundance of SEPT5 proteins at the area of infection.

### 3.3. SEPT5 Protein Expression in E. coli

**Figure 5:**
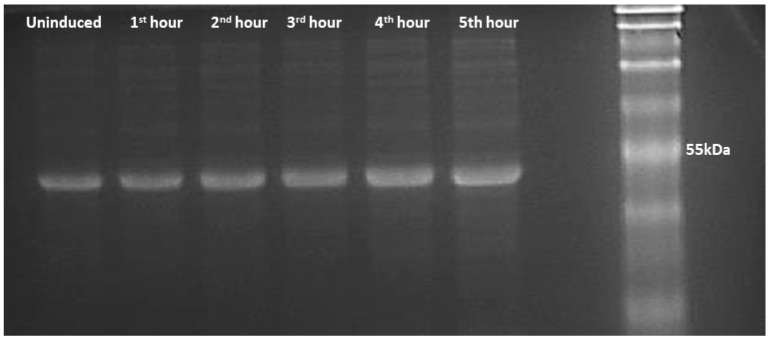
Recombinant SEPT5 protein expressed in BL21 (DE3) cells from pRSETB vector. SDS-PAGE photo of expressed proteins from cell pellets, before and after induction by IPTG. The arrow indicates uninduced expression followed by hourly expression until the fifth hour of bacterial culture collection. The size of the protein lies between 40 and 55 kDa.

**Figure 6:**
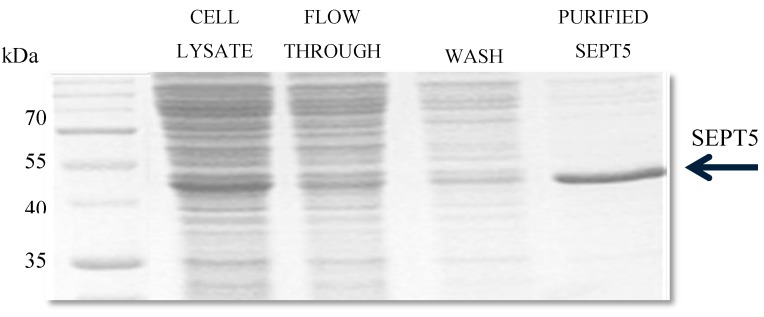
Purification of recombinant SEPT5 protein expressed in *E. coli*. SDS-PAGE of bacterially-expressed recombinant prepared through Ni-NTA column after induction of *E. coli* with IPTG for 18 h at 16 °C in lysate, flow through, wash fraction and, finally, the purified protein was obtained at 46 kDa.

**Figure 7:**
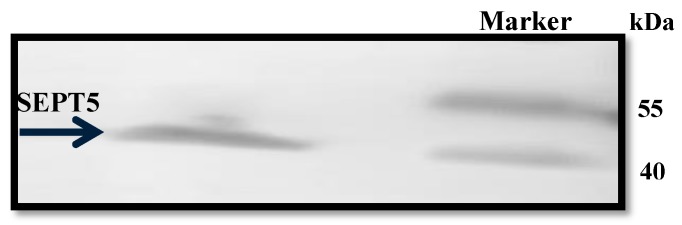
Detection of recombinant SEPT5 with anti-SEPT5 antibodies. The purified SEPT5 was resolved in SDS-PAGE and transferred to a PVDF membrane for detection with Western blotting using an anti-SEPT5 polyclonal antibody (Abcam, Cambridge, MA, USA). A single protein band was observed between 45 and 55 kDa molecular weight.

A plasmid of the correct sequence alignment of the gene was used for SEPT5 protein expression. Protein expression was performed in BL21 (DE3) competent cell lines for 5 h (Figure 5). SEPT5 was purified on a His-tag Ni-NTA chelating column and the yield of purified proteins was ~2.5 mg of SEPT5 per litre of induction. As shown in Figure 6, the recombinant protein migrated in SDS–PAGE to bands corresponding to molecular weights of ~50 kDa which are in agreement with the predicted values of 46 kDa. Western blot was performed to detect the antigenicity of the purified protein using α-SEPT5 (Figure 7) where the purified recombinant proteins were able to be detected with the commercial polyclonal antibodies. Validation of expressed proteins as SEPT5 was done with LCMS/MS where it detected distinctive peptides corresponding to SEPT5 *Gallus gallus* proteins with 45% amino acid coverage.

### 3.4. The rSEPT5 Protein Was in Its Conformational State

**Figure 8:**
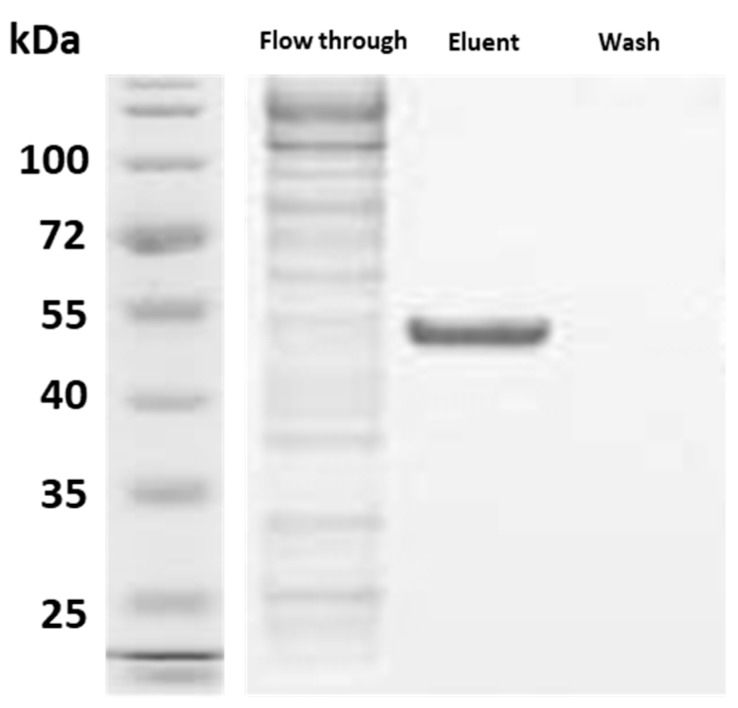
Detection of recombinant SEPT5 immunoprecipitated with polyclonal anti-SEPT5 in SDS-PAGE. Anti-SEPT5 polyclonal antibodies (Abcam, Cambridge, MA, USA)-Protein A/G agarose beads complex were added to the purified rSEPT5 protein and incubated at 4 °C overnight. After stringent washing, he sample buffer was added to the antibody-protein complex and run in SDS-PAGE. The protein band detected in the eluent fraction was between 45 and 55 kDA which were the bound rSEPT5 proteins while the flow-through fraction contained unbound proteins.

The recombinant expression of SEPT5 proteins induced the formation of soluble proteins in the solution which conserved sufficient features for being recovered by affinity chromatography. These proteins were then able to be captured by the polyclonal antibody which was an indication of proper protein folding. Figure 8 shows the rSEPT5 that was captured by the polyclonal anti-SEPT5 in the eluent fraction from the immunoprecipitation assay.

### 3.5. Septin Proteins Interact with Various Host’s Proteins in Infected Brain Lysates

**Figure 9:**
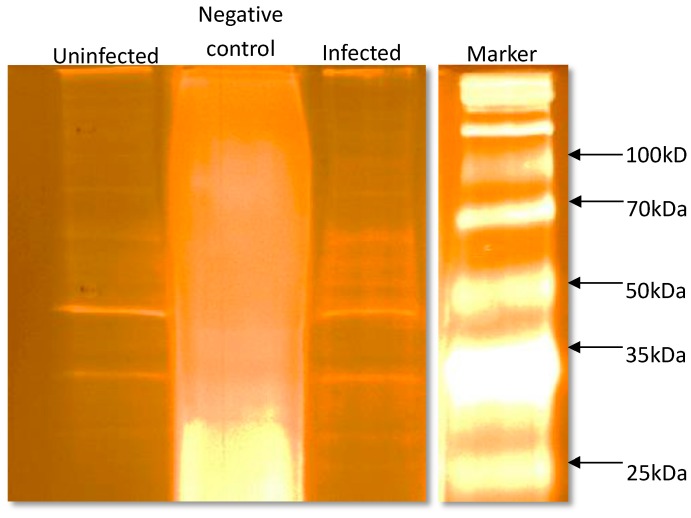
Co-IP of proteins from chicken brain lysates as observed with silver stain. Silver staining of eluted protein complexes of uninfected and infected brain samples developed intense colour for detected proteins. Protein bands were excised for in-gel digestion and identification with MS/MS.

To determine the interacting proteins of SEPT5 in neuronal cells during H5N1 infection, co-IP assays were performed with the brain lysates. Non-infected and infected lysates were pulled down with commercially-available SEPT5 polyclonal antibodies that were bound to immobilize resin. The eluted fractions for brain lysates were subjected to silver staining as low protein abundance was observed in Coomassie Blue staining (Figure 9). Bands were then excised and digested with trypsin to determine its identity by MS/MS. In infected brain tissue lysates, more host’s proteins, including other septin families, were eluted out (Table 2) compared to the non-infected control (Table 3). Apart from other host’s proteins being pulled down, rSEPT5 protein was also eluted out from the immunocomplexes in both infected and non-infected lysates, which indicated that the proteins successfully captured and bound to the polyclonal antibodies. The authenticity of the rSEPT5 and the correct confirmation of the septin protein was confirmed when anti-SEPT5 polyclonal antibodies were able to pull down rSEPT5 with other rSEPT5-bound host proteins from the lysates.

## 4. Discussion

Highly-pathogenic H5N1 influenza A viruses can cause fatal systemic infection in poultry. The HPAI virus replicates primarily in endothelial cells of the vascular systems and spreads to other body systems and penetrates the blood brain barrier to infect the neurons and glial cells of the brain [59]. However, their neurotropism and effects on the central nervous system (CNS) are not fully understood. We assessed H5N1 viral invasion of the CNS in an infected chicken brain model which are highly susceptible to infection with influenza viruses. The H5N1 virus caused spongiosis in brain tissues and oedema. Similar signs and symptoms have also been shown to develop in humans infected with virulent H5N1 avian influenza viruses [60,61,62]. These data suggest that this H5N1 virus could cause neurological effects on the CNS in chickens.

SEPT5, a known parkin substrate, is a vesicle and membrane associated protein that plays a significant role in inhibiting exocytosis in the neurons [16]. In a recent study by Balasubramaniam et al., SEPT5 was recently shown to be upregulated in chicken brain infected with H5N1 Avian Influenza virus, indicating the possible role played by this protein in the pathogenesis of influenza [24]. Our current study has also shown the localization and possible interactions of the influenza virus with SEPT5 in chicken brain tissues with abundant SEPT5 proteins observed in infected tissues and localized in the substantia nigra. In a study in the mouse brain, SEPTf5 is expressed in presynaptic axon terminals where it is densely distributed near synaptic vesicles [63]. As SEPT5 is abundantly expressed in the brain, this suggests an association in the pathogenesis of the virus in the host’s CNS.

Our understanding of septin function depends on knowledge of the biochemistry of these proteins. However, their study has been hampered by difficulties associated with their expression as recombinant proteins in bacteria and the complexity of their interactions with one another. Limited studies have reported the production of septin proteins in any expression system. In this report, we present a protocol of cloning, expression, and purification of *Gallus gallus* SEPT5 protein in *E. coli*. The ORF coding sequence of chicken SEPT5 was cloned into the pRSETB expression vector and transformed in *E. coli* BL21 (DE3) cells. This protocol yields a considerable amount of protein under optimized conditions and was proven to be authentic.

To improve the soluble fraction of pRSETB/SEPT5, low-temperature cultivation of bacteria was utilized, which is a well-known technique to limit the in vivo aggregation of recombinant proteins. This strategy has been proven effective in improving the solubility of a number of difficult proteins, including interferon α-2, subtilisin, ricin A chain, β-lactamase, and rabbit muscle glycogen phosphorylase [64]. Studies by Maimaitiyiming et al. [65] have established a method to express and purify various septin proteins using the *E. coli* expression system at low temperature. Their study captured the shape of the septins to be of single or parallel thin filaments, as has been reported by their predecessors [66,67,68]. It was then concluded that the low temperature was beneficial to folding and the system was suggested as a tool for expression and correct folding of recombinant SEPT5 in the cytoplasm of *E. coli.* This is the first study of chicken SEPT5 protein that was successfully expressed in its conformational form in *E. coli*.

In the influenza virus replication cycle, the focus was mainly on host factor interactions, especially with viral ribonucleoproteins (vRNPs) or of the polymerase by using affinity purification or yeast two-hybrid techniques [69,70]. A proteome-wide screen of virus-host protein-protein interactions has also provided an important resource of 135 interactions. This study, however, highlighted major cellular functions that are essential for virus replication. Both cytoplasmic and membrane-bound proteins were identified as interacting partners with SEPT5 through pull-down co-IP assay in infected lysates. Many proteins are related or can be grouped together in functional categories, such as cellular cytoskeletal components and enzymes. It has been reported that cytoskeleton proteins and cytoskeleton-associated proteins provide numerous support functions for viral gene expression to allow an infectious cycle [71]. For influenza, an intact actin cytoskeleton is required for viral entry [72] and both actin and tubulin were identified as proteins that interact with influenza virus RNPs [73]. Beta-actin was also present in the interior of the influenza virions, which most likely reflects their active participation in moving the viral components to the assembly site, as well as re-organization of cytoskeleton [74].

The CRMP2/DPYSL2 protein is a CNS protein involved in neuronal development, axonal and neuronal growth, cell migration, and protein trafficking. CRMP2 is known to function as a microtubule-binding protein with structural and regulatory roles in cytoskeletal dynamics, vesicle trafficking, and synaptic transmission in the developing brain and in cell cultures [75,76]. One of the best-characterized roles of CRMP2 in the developing brain is the regulation of synaptic dynamics. Martins and colleagues have identified one of the interacting proteins of coiled-coil CRMP2 containing protein kinase 2 (ROCK2) [77]. ROCK2 and other septins, such as SEPT3, SEPT6, and SEPT9, have been implicated as components of the rho signaling pathway, which is comprised of a variety of rho GTPases. This family of proteins was one of the most represented in the pathway analyses of the CRMP2 interactome. CRMP2 was also upregulated in the CNS of G*allus gallus* upon infection with HPAIV, but was not upregulated in uninfected brain tissues [33].

GAPDH was the first glycolytic enzyme found associated with tubulin [78,79]. In Japanese encephalitis virus infection, GAPDH subcellular localization was changed by interacting with viral RNAs and co-localized to the NS5 protein, suggesting a role during virus life cycle [35].

Hsps are a diverse group of highly conserved and ubiquitous cytoprotective proteins that are divided, by convention, into different classes according to molecular size, e.g., Hsp100 (100 kDa molecular mass), Hsp90 (90 kDa), Hsp70 (70 kDa), Hsp60 (60 kDa), Hsp40 (40 kDa), and the small Hsps (9–43 kDa). During influenza virus infection, Hsp90 interacts with the viral RNA-dependent RNA polymerase, playing a role in the assembly and nuclear transport of viral RNA polymerase subunits en route to the formation of a mature polymerase complex [80,81]. Hsp90 is also a binding partner of NS1 of H5N1 influenza virus which mediates cell apoptotic response by activating a caspase cascade [82]. Association of heat shock cognate protein 71 (Hsc71) in infected lysates may be due to the fact it is required for optimal polymerase function in infected cells and the protein function in cellular stresses [83]. Hsc71, which is a constitutive form of the Hsp70 family of proteins was previously reported to bind to influenza virus matrix 1 protein in infected cells and resulted in an inhibition of vRNPs export from the nucleus, thus causing a reduction of virus production. Hsc71 is, therefore, required for the late stage of virus infections [84]. However, for the majority of identified host factors from brain lysates, the mode of action remains to be determined.

## 5. Conclusions

This is the first study to have successfully cloned the *Gallus gallus* SEPT5 construct and expressed the recombinant protein in a soluble fraction using *E. coli* cells. The recombinant protein constituted by the GTPase domain and C-termini of *Gallus gallus* SEPT5 was expressed and purified in large scale quantities and confirmed by mass spectrometry. The recombinant protein is folded in its proper form and stable, as shown by detection and pull-down of the rSEPT5 protein using a commercial polyclonal SEPT5 in a co-IP, by Western blot and SDS-PAGE assays. To date, knowledge of the details of the expression array of septin transcripts and isoforms in human cells and tissues, and the regulatory events, are few and variable. Previously, a low-resolution analysis by expression microarray has been reported and published along with surveys of other septins [85,86,87]. With regard to some septins, those data have been validated by RT–PCR and limited antibody-based methods [88,89]. In other cases, septin involvement in the expression and perturbation in infectious diseases are not entirely known. As with the full definition of the details of septin genomics and transcriptomics, there is an urgent need for a comprehensive analysis of septin expression profiles. Nevertheless, it is clear that there are profound differences in the expression of septins across human tissues and in disease states. We would contend that defining the details of septin transcript expression, the subcellular distribution and stoichiometries of the various isoforms will be an essential step towards developing a full understanding of septin biology.

## Figures and Tables

**Table 1:** Sequence of the SEPT5 gene.

Accession No.	NP_001025825.2
Gene	SEPT5 (*Gallus gallus*)
Protein	Septin 5
Forward Primer	GCC TCG AGG *Eco*RI ATG AGC ACC GGC ACG CGC TAC AAG AGC AAG CCG CTC AAC CCA GAG GAG AAG CAG GAC
Reverse Primer	AGA AGC TTC *Hind*III TAG TCA CTG ATC CTG CAT CTG CTG CTG CAT CTT
Amplicon Length	1110 bp
Protein Size	45 kDa

**Table 2:** List of identified interacting proteins from H5N1-infected brain lysates.

Proteins	Functions	Accessiona	References
Actin filaments, cytoplasmic 2	Major constituent of cytoskeleton, actin filaments are also involved in cytokinesis and cell movement	NP_001295542.1	[29,30]
Dihydropyrimidinase-related protein 2 (DPYSL-2/CRMP2)	Involved in neuronal development, axonal and neuronal growth. May also be involved in viral pathogenesis	NP_001184222.1	[31,32,33,34]
Glyceraldehyde-3-phosphate dehydrogenase (GAPDH)	Involves in glycolysis; the enzyme has been found to bind to actin and tropomyosin, and may thus have a role in cytoskeleton assembly	AAA48774.1	[35,36]
Heat shock cognate 71 kDa protein (Hsc71)	Regulates stress response and induced inflammatory response, including TNF secretion	NP_990334.1	[37,38]
SEPT2	Required for normal organization of actin microfilament. May also play a role in the internalization of *Listeria monocytogenes* and *Shigella flexneri* and anti-viral response to *Vaccinia virus*	NP_001006182.1	[39,40,41]
SEPT5	Filament forming cytoskeleton GTPase, involved in Parkinson’s pathogenesis and may regulate exocytosis and cell division	NP_001025825.2	[19,42,43,44]
SEPT6	Required for actin assembly	NP_001026296.1	[17,45,46]
SEPT7	Required for normal actin organization	NP_00102577.1	[47,48,49,50]
PREDICTED: SEPT11 isoform	May play a role in cytoarchitecture of neurons including dendritic arborization and the formation of phagosome. May also be involved in anti-viral response to *Vaccinia virus*	XP_004941218	[51,52,53]
Tubulin β-3	Proper axon guidance and maintenance	NP_001074329.2	[54]
Tubulin β-7	GTPase binding major constituent of microtubule	NP_990646.1	[55]

^a^ Data gathered from NCBI protein database.

**Table 3:** List of identified interacting proteins from non-infected brain lysates.

Proteins	Functions	Accession ^a^	References
Actin filaments, cytoplasmic 2	Major constituent of cytoskeleton, actin filaments are also involved in cytokinesis and cell movement	NP_001295542.1	[29,30]
SEPT5	Filament forming cytoskeleton GTPase, involved in Parkinson’s pathogenesis and may regulate exocytosis and cell division	NP_001025825.2	[19,42,43]
SEPT7	Required for normal actin organization	NP_00102577.1	[47,48,49,50]
Tubulin α	Structural constituent of cytoskeleton	CAA30852.1	[56,57]
Tubulin α-5	A major constituent of microtubule	P09644.1	[58]
Tubulin β-7	GTPase binding major constituent of microtubule	NP_990646.1	[55]

^a^ Data gathered from NCBI protein database.
